# Expérience de réorganisation d'une maternité de troisième niveau face à la pandémie de la COVID-19: étude du cas de la maternité de l´hôpital universitaire de Marrakech

**DOI:** 10.11604/pamj.2022.41.38.26186

**Published:** 2022-01-13

**Authors:** Hajar Ouahid, Latifa Adarmouch, Abderraouf Soummani, Mohamed Cherkaoui, Majda Sebbani, Mohamed Amine

**Affiliations:** 1 Faculté de Médecine et de Pharmacie, Université Cadi Ayyad, Laboratoire Bioscience et Santé, Marrakech, Maroc,; 2 Centre Hospitalier Universitaire Mohammed VI, Service de Gynécologie Obstétricale, Marrakech, Maroc,; 3 Faculté de Médecine et de Pharmacie, Université Cadi Ayyad, Département de Santé publique, Médecine Communautaire et Epidémiologie, Marrakech, Maroc,; 4 Centre Hospitalier Universitaire Mohammed VI, Service de Recherche Clinique, Marrakech, Maroc,; 5 Faculté des Sciences Semlalia, Université Cadi Ayyad, Laboratoire de Pharmacologie, Neurobiologie, Anthropobiologie et Environnement, Marrakech, Maroc

**Keywords:** Pandémie, maternité de l’hôpital, COVID-19, Pandemic, maternity hospital, COVID-19

## Abstract

**Introduction:**

la pandémie de la COVID-19 a entraîné des pressions sans précédent sur les services de soins en gynéco-obstétrique. Les maternités devaient se préparer à fournir des soins de qualité tout en empêchant la transmission de l'infection. Cette étude avait pour objectif de décrire les éléments clés de la réponse à la COVID-19 au sein de la maternité du CHU Mohammed VI de Marrakech.

**Méthodes:**

un plan d'étude de cas a été adopté. Les données ont été collectées à travers l´exploitation des différents documents administratifs intéressant l´activité de la maternité du CHU Mohammed VI, durant la pandémie. Nous avons également mené des entretiens semi-structurés avec des professionnels au niveau de la maternité. Une méthode d'analyse thématique qualitative a été utilisée pour l'analyse des données retranscrites et la triangulation avec l´analyse des données documentaires.

**Résultats:**

l´étude a mis en exergue les étapes de conception et d´organisation de deux circuits d´accès de la maternité. Un nouveau circuit pour les patientes suspectes ou confirmées COVID-19 a permis d´assurer l´accès à une prise en charge de qualité des patientes en garantissant leur isolement. Le circuit habituel est resté fonctionnel pour consolider le droit d´accès aux soins gynéco-obstétricaux tertiaires tout en appliquant les mesures de protection contre la COVID-19.

**Conclusion:**

il est nécessaire de s´inspirer de l´expérience de préparation des autres établissements de santé, de contextualiser les actions locales et d´anticiper l´organisation du travail face aux crises sanitaires.

## Abstract (en)

**Introduction:**

COVID-19 pandemic has put unprecedented pressures on obstetrics and gynecology care services. Maternity hospitals have needed to rapidly prepare to provide quality care while preventing the transmission of the infection. The purpose of this study is to describe the key elements of the response to COVID-19 within the maternity ward of the Mohammed VI University Hospital in Marrakech.

**Methods:**

a case study was conducted. Data were collected using various administrative documents related to activities in the maternity ward of the Mohammed VI University Hospital during the COVID-19 pandemic. We also conducted semi-structured interviews with professionals at the maternity ward. Qualitative thematic analysis approach was used for the analysis of transcribed data and triangulation, with the analysis of documentary data.

**Results:**

the study highlighted phases of design and organization of two maternity ward access circuits. A new circuit for suspicious or confirmed cases of COVID-19 ensured access to quality care for patients, by guaranteeing isolation measures. The access circuit that was commonly used remained functional to consolidate the right of access to tertiary obstetrics and gynecology care while applying protective measures against COVID-19.

**Conclusion:**

this study highlights the necessity to draw on the experience of other health facilities, to contextualize local activities and to anticipate work organization in the face of health crises.

## Introduction

Le nouveau coronavirus est une infection apparue en novembre 2019, répandue comme cause de pneumonie virale et déclarée pandémie mondiale [1]. La pandémie de la COVID-19 est exceptionnelle en raison de son ampleur, de sa vitesse de propagation, de sa gravité et de manque de données scientifiques préexistantes [2]. Au Maroc, parmi les régions les plus touchées par ce virus, on trouve la région de Marrakech-Safi [3].

La capacité des hôpitaux à se réorganiser, à maintenir les fonctions essentielles et répondre aux urgences sanitaires, est primordiale [4,5]. Conscient de cette situation, le Maroc, à l´instar d´autres pays, a instauré plusieurs stratégies en urgence visant à réduire la propagation du nouveau coronavirus, et la prise en charge des personnes atteintes de ce virus [6]. Ces dernières années, le Maroc a connu une réduction importante de la mortalité maternelle et néonatale [7]. Le défi relevé par les maternités du royaume était de consolider ce progrès durant cette crise sanitaire et de maintenir un accès aux soins obstétricaux, sécurisé et de qualité.

Le Centre Hospitalier Universitaire (CHU) Mohammed VI de Marrakech est devenu une structure de référence pour le dépistage et la prise en charge des cas confirmés de COVID-19 [8]. La maternité du CHU Mohammed VI de Marrakech représente la seule institution publique dans la région Marrakech-Safi fournissant une gamme de soins tertiaires en gynéco-obstétrique [9]. Le détournement des ressources de soins de santé pour contenir la pandémie de la COVID-19 a considérablement nui à l'accessibilité et à la disponibilité des services de santé essentiels. La pandémie du coronavirus est spécifique vu le risque de contagion rapide, le flux de patientes à gérer, le respect des barrières de protection, et surtout le profil spécifique de ces patientes qui sont plus à risque face aux infections virales [10]. Elles ont un risque accru de développer une forme sévère de la maladie liée à la COVID-19 [11].

Notre étude avait comme objectif de décrire les éléments clés de la réponse à la COVID-19 au sein de la maternité CHU Mohammed VI de Marrakech, y compris les défis pour maintenir l´accès et les problèmes à anticiper suite à l'expérience de cette maternité. Ces données sur l´organisation et le circuit des patientes face à la pandémie de la COVID-19 pourrait servir de modèles et de leçons dans des situations et contextes similaires.

## Méthodes

### Conception et cadre de l'étude

**Conception de l'étude:** nous avons réalisé une étude de cas relatant la réponse de la maternité du CHU Mohammed VI à la pandémie.

**Cadre de l'étude:** la maternité du CHU Mohammed VI de Marrakech représente la seule institution publique dans la région Marrakech-Safi fournissant une gamme de soins tertiaires en gynéco-obstétrique. Cette structure a maintenu la prise en charge des urgences maternelles et infantiles [10].

### Collecte de données

**Les instruments utilisés pour collecter des données:** les données ont été collectées à travers l´exploitation des différents documents administratifs intéressant l´activité de la maternité du CHU Mohammed VI durant cette période. Aussi d´autres données ont été collectées par entretiens semi-structurés, avec des sage-femmes, des obstétriciens, des infirmières, des majors des services et des surveillants.

**Le processus global de collecte de données:** nous nous sommes intéressés au circuit de prise en charge des urgences gynéco-obstétricales, salle d´accouchement, bloc opératoire, jusqu´à l´hospitalisation dans les services des suites de couches et des grossesses à haut risque. Ainsi que le nouveau service d´hospitalisation COVID-19. La période de collecte des données était du 20 avril au 20 mai 2020. Nous avons utilisé deux sources de données, les entretiens semi-structurés avec les professionnels et l´analyse des documents administratifs, circulaires ministériels, notes de service concernant l´organisation du nouveau circuit, la gestion du flux, les procédures de dépistage et de prise en charge des cas suspects et des cas confirmées ayant la COVID-19 ainsi que les documents intéressant le management et la prise en charge des professionnels de santé en contact direct avec les patients ayant l´infection à COVID-19. Le guide d´entretien était élaboré par les auteurs et testé par les membres du laboratoire de recherche.

### Entretiens

**Les participants cibles:** les participants interviewés étaient sélectionnés par choix raisonné. C´étaient des sage-femmes, des obstétriciens, des infirmières polyvalents qui assuraient les prestations de soin au profit des patientes entrant dans les deux circuits adoptés, ainsi que les majors des services et les surveillants généraux qui assuraient la gestion des différents services concernés. Dix professionnels de santé ont participé à cette étude.

**Déroulement des entretiens:** les entretiens étaient réalisés en face à face et un enregistrement vocal a été effectué. Un consentement était signé par chacun des participants après explication de l´objectif de l´étude étant le partage d´expérience en cette situation pandémique permettant aux autres établissements de s´inspirer, éviter les obstacles et anticiper. Une relation de confiance et du respect de la confidentialité était établi. Le guide d´entretien a compris deux grandes questions intéressantes: les dispositions mises en place pour faire face à la pandémie du coronavirus et les perceptions et recommandations des participants. Des questions de relance ont été utilisées lorsque les réponses n´étaient pas précises ou claires. Les professionnels ont été choisis en raison de leurs rôles dans le circuit de prise en charge. Les profils étaient diversifiés pour avoir le maximum d´information jusqu´à la saturation des données. Aucune autre personne n´était présente durant l´entretien. La durée moyenne des entretiens était de 25 minutes. Les entretiens étaient conduits par l´auteur correspondant. C´était une sage-femme ayant un master de recherche en santé publique et en étant doctorante au laboratoire de recherche.

**Synthèse des données:** une analyse qualitative a été réalisée sur les entretiens transcrits ainsi que les documents. Nous avons utilisé l'analyse de contenu thématique [12]. Nos thèmes ont été identifiés au niveau des entretiens et la triangulation a été faite avec l´analyse des données documentaires. Trois chercheurs ont participé au codage des données. Nous avons commencé par la transcription mot par mot des données de l´enquête. Ensuite, nous avons entrepris une lecture active des données transcrites, tout en recherchant les significations et les thèmes. Nos données étaient codées autour de questions spécifiques. Ensuite, les différents codes pertinents ont été triés pour identifier des thèmes pertinents.

**Considérations éthiques:** les principes de l´éthique de la recherche ont été respectés. La confidentialité et l´anonymat des participants a été respecté lors de la collecte et de l'analyse des données. Le consentement éclairé a été obtenu par les participants et enregistré en début de l´entretien. La participation était libre et volontaire. Selon la loi marocaine sur la bioéthique, l'opinion du comité d'éthique n'a pas été demandée puisqu'il s'agit d'expression d'opinion sans intervention humaine [13]. Aussi, une autorisation pour l´exploitation des documents administratifs a été obtenue avant le démarrage de l´étude.

## Résultats

**Figure 1:**
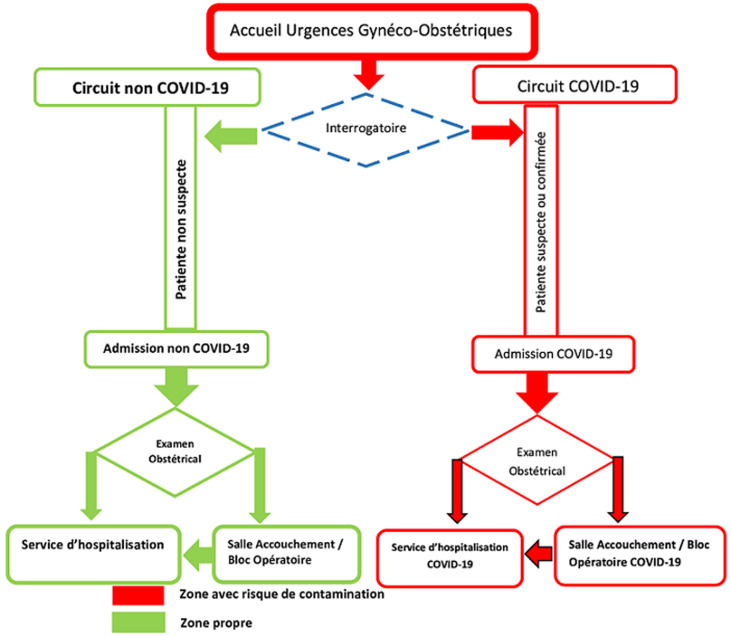
organisation du circuit d´accès de la maternité du CHU Mohammed VI de Marrakech pendant la pandémie de la COVID-19

**Figure 2:**
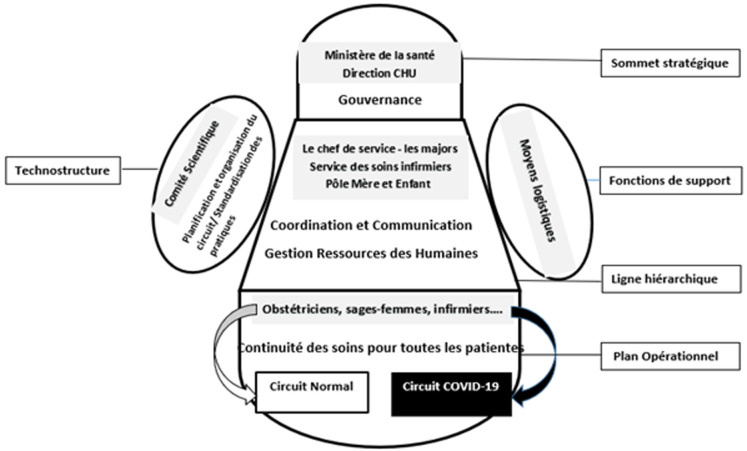
structure organisationnelle de la maternité durant la pandémie de la COVID-19

L´analyse des documents et des entretiens a dévoilé la nouvelle organisation et les axes de préparation face à la pandémie de la COVID-19 dans la maternité CHU Mohammed VI (Figure 1). Concernant les entretiens, dix professionnels de santé ont participé à cette étude. La moyenne de l'âge était de 33,0 ± 4,2 ans. Le premier thème ressorti des données intéressait les éléments d´organisation des deux circuits de soins (Figure 2). Les autres thèmes mettaient en évidence l´organisation des professionnels, la communication et la gestion des moyens logistiques.

**L´organisation des circuits d´accès durant la pandémie de la COVID-19:** après création d´un comité scientifique au sein de la maternité, une nouvelle organisation du circuit d´accès a été adoptée. Ce nouveau circuit avait comme objectif de séparer les patientes suspectes ou confirmées COVID-19 des autres patientes non suspectes [14,15]. “*Quel que soit le motif de consultation de la patiente aux urgences, une hôtesse d´accueil administre en premier un questionnaire pour dépister si elles avaient des anomalies suspectes à fin de rejoindre le circuit COVID-19”* (sage-femme 2). La salle d´admission était divisée en deux salles, une pour les cas suspects et l´autre pour les cas non suspects. Les éléments du diagnostic et de prise en charge des patientes ont été détaillés et affichés [15,16]. “*Des fois l´interrogatoire approfondi avec la patiente suspecte pouvait éliminer l´infection à COVID-19 et attribuait par exemple une fièvre à une autre cause comme une pyélonéphrite aiguë, dans ce cas la patiente rejoint le circuit normal”* (obstétricien 1). Si l´entretien révèle toujours une symptomatologie suspecte, un prélèvement PCR est fait. “*Si l´état de la patiente présentait des signes d´aggravation on faisait appel au réanimateur pour une prise en charge à la réanimation COVID-19”* (obstétricien 2).

La salle d´accouchement et la salle du bloc opératoire étaient isolées et désinfectées avant et après le passage des patientes. *Dès qu´on termine l´accouchement normal ou la césarienne, la patiente suspecte et son nouveau-né étaient acheminés au service d´hospitalisation COVID-19 pour bénéficier d´une surveillance et d´un confinement* (obstétricien 2). Pour gérer l´encombrement routinier des urgences un numéro de téléphone attribué au SAMU du CHU a été lancé, dans un but de diminuer les consultations non urgentes. *Nous avons essayé d´augmenter la capacité litière à travers l´aménagement des salles de cours et bureaux* (major 2). Le temps d´hospitalisation en post partum normal avait été réduite selon chaque cas. Aussi la restriction du nombre de visiteurs [15]. Malgré ces efforts, une saturation de la capacité litière avait été signalé. *Le flux d´accouchement était augmenté un certain moment à cause de la fermeture d´autres maternités à Marrakech à cause de la contamination de personnel soignant*(obstétricien 1).

**Préparation des professionnels de santé:** l´analyse des documents a révélé la nouvelle stratégie de déploiement du personnel de soins en système de garde pour assurer la continuité des soins avec respect du temps de repos réparateur. Ainsi que la suspension des congés [17]. Les femmes enceintes et les professionnels souffrant de maladies chroniques ont été exclus de tout contact avec les patients atteints de la COVID-19 et un système d´autosurveillance pour le suivi et la prise en charge des professionnels de santé avait été établie [15-17]. “*La première des choses à réaliser était de récupérer tout le personnel à disposition et d´anticiper la réorganisation de l´horaire de travail* (surveillant 1).

La discussion sur la formation avec les professionnels sur la prise en charge des cas COVID-19 et l´utilisation des moyens de protection a montré des opinions divergentes. “*Les vidéos que nous avons reçues ne s´adaptent pas à notre contexte, surtout que les moyens physiques utilisés dans ces vidéos ne sont pas disponibles dans notre hôpital”* (sage-femme 2). La plupart des interviewés ont souligné l´impact de cette crise sanitaire sur leur état émotionnel. “*On se sentait en danger chaque instan”t* (infirmier 1).

**Coordination et communication:** la coordination était un pilier important à maintenir durant cette crise. “*Toutes nos communications étaient par email. Nous avons essayé d´éviter au maximum les courriers qui peuvent être source de transmission du virus entre les services”* (Major 2).

**Moyens logistiques:** l´identification détaillée des moyens de protection et de leur approvisionnement était précisée dans plusieurs documents [15]. La plupart des participants ont affirmé que la disponibilité des moyens de protection physique a connu une amélioration après le début de la pandémie. ”*On avait des problèmes de dotation de bavettes pour se protéger au début de la pandémie, surtout que ces bavettes devaient être changées chaque 3 heures”* (sage-femme 2).

## Discussion

Cette étude nous a permis d'explorer la nature des actions d'organisation du travail dans la maternité du CHU Mohammed VI de Marrakech face à la pandémie de la COVID-19. La préparation a compris plusieurs aspects de l´organisation du circuit d´accès des patientes suspectes ou confirmées COVID-19. Nos résultats ont montré que la préparation des professionnels était d´assurer leurs dispositions, et admettre une nouvelle répartition d´horaire de travail des équipes de soins. D'autre part, nos résultats ont montré que la formation continue avec des données actualisées et standardisées des professionnels de santé est un volet important pour améliorer la prise en charge des patientes.

Une conduite similaire est reportée en Italie et en Roumanie. Les auteurs ont adopté aussi une nouvelle organisation du circuit d´accès des patients COVID-19 [18,19]. Ils ont formé le personnel à la gestion des cas COVID-19, l´organisé en équipes distinctes; chaque équipe travaillant pendant 6 à 9 jours, les autres équipes étant restées en attente d'entrer lorsque le nombre de cas COVID-19 augmentera [19]. De même, d´autres études ont montré que la collaboration et la formation conjointe entre les différentes équipes cliniques impliquées dans la prise en charge de ces patients s'est avérée être l'un des moyens les plus efficaces pour améliorer les performances [20-22].

La prise en charge sûre et optimale de la parturiente pendant la période péri-partum nécessite une approche d'équipe multidisciplinaire, surtout si la parturiente est atteinte du COVID-19 [23]. La pneumonie résultant de toute étiologie infectieuse est une cause importante de morbidité et de mortalité chez les femmes enceintes. C'est la maladie infectieuse non obstétricale la plus répandue qui survient pendant la grossesse [24-26]. Dans le cadre d´une série d´études, elle s´est avérée être la troisième cause en importance de décès obstétrical indirect [27]. Les résultats de notre étude ont montré qu´il était nécessaire d´assister toute patiente suspecte ou confirmée COVID-19, d´assurer une prise en charge complète et d´éviter les complications liées à cette infection selon les protocoles établis.

En parallèle de l´activité COVID-19, nos résultats ont souligné l´importance de maintenir l´accès sécurisé de la maternité pour les patientes non COVID-19. Les participants ont soulevé le danger que peuvent encourir les patientes qui ont des cancers gynécologiques si leurs prestations de soins tardent, ainsi que le risque pour les patientes avec des grossesses à haut risques nécessitant des soins des niveaux tertiaires. De même, dans une étude sur l´impact de la pandémie du coronavirus sur la pratique chirurgicale, les auteurs ont souligné l´importance de développer un espace opérationnel dédié au COVID-19, dans le cadre d'une intervention plus large et de maintenir l´activité essentielle de l´hôpital pour les autres patients [28].

La variabilité de la disponibilité des moyens de protection pour le personnel a créé beaucoup de soucis durant la pandémie. La COVID-19 est extrêmement transmissible, chaque cas ensemençant plus de deux cas secondaires [29,30]. Dans le rapport de la mission conjointe OMS-Chine, 2055 agents de santé représentaient 3 à 7% des cas avec COVID-19 confirmés en laboratoire en Chine [31]. L'OMS recommande que les équipements de protection individuels pour les agents de santé fournissant des soins directs aux patients atteints de COVID-19 comprennent des masques médicaux, des blouses, des gants et une protection oculaire [32].

La plupart des interviewés ont réclamé aussi l´atteinte de leurs état émotionnel durant cette crise sanitaire. Des degrés sévères de stress et des symptômes de dépression et d'anxiété ont été trouvés chez 2,2% à 14,5% des participants dans une étude sur le stress psychologique des travailleurs de la santé causé par la pandémie de la COVID-19 [33]. Cependant, les résultats d´une autre étude sur la prévalence de la dépression et de l'anxiété autodéclarées parmi les membres du personnel médical pédiatrique lors de l'épidémie de la COVID-19 à Guiyang en Chine, ont montré que les répondants qui avaient une expérience d'exposition ont rapporté des taux plus élevés d'anxiété accompagnée de dépression que les répondants qui n'avaient aucune expérience d'exposition (taux d'incidence de 31,6% et 12,6%, respectivement; χ^2^= 4,1, p = 0,042) [34].

Notre étude a certaines limites. Celle-ci a été menée dans une seule maternité à cause des difficultés d'accès aux professionnels de santé des autres maternités en raison de la restriction de la mobilité entre les établissements de soins pour risque de contamination par la COVID-19. En revanche, le choix de la maternité du CHU était intéressant vu que c´est une institution de référence du niveau de soins tertiaires et la seule maternité de la région de Marrakech-Safi qui a assuré aussi bien l´activité COVID-19 en parallèle avec son activité normale. D´un autre côté, le choix des profils des professionnels était diversifié pour avoir le maximum d´information et la saturation des données. De plus, nous avons appliqué différentes méthodes de collecte de données et effectué une triangulation pour améliorer la crédibilité des résultats.

## Conclusion

Cette étude a mis en exergue les étapes de conception et d´organisation de deux circuits d´accès de la maternité. Un nouveau circuit pour les patientes suspectes ou confirmées COVID-19 a permis d´assurer l´accès à une prise en charge de qualité des patientes en garantissant leur isolement. Le circuit habituel est resté fonctionnel pour consolider le droit d´accès aux soins gynéco-obstétricaux tertiaires tout en appliquant les mesures de protection contre la COVID-19. Les leçons apprises de cette expérience seront d´un grand intérêt pour les autres établissements de santé à travers le monde afin de s´inspirer, de contextualiser les actions locales et d´anticiper. Plusieurs recommandations ont été proposées à savoir l´augmentation des ressources allouées aux hôpitaux, la formation et l´organisation des professionnels de santé et la nécessité d´une implication multidisciplinaire et à plusieurs niveaux vers un objectif commun: assurer un accès à des soins sécurisés et de qualité.

### Etat des connaissances sur le sujet

- *L´intérêt de l´anticipation et la préparation des hôpitaux face aux crises sanitaires;*
- *L´importance de la prévention pour limiter la propagation du virus de la COVID-19*.

### Contribution de notre étude à la connaissance

- *Les éléments clés de la conception et la préparation de cette maternité, que les autres établissements peuvent s´en inspirer pour anticiper la préparation face aux crises sanitaires;*
- *Le vécu des professionnels de santé durant cette pandémie*.
